# A Hedonism Hub in the Human Brain

**DOI:** 10.1093/cercor/bhw197

**Published:** 2016-09-19

**Authors:** G. Zacharopoulos, T. M. Lancaster, T. Bracht, N. Ihssen, G. R. Maio, D. E. J. Linden

**Affiliations:** 1 CUBRIC, School of Psychology, Cardiff University, Cardiff, Wales, UK; 2 MRC Centre for Neuropsychiatric Genetics and Genomics, Cardiff, UK; 3 National Centre for Mental Health, Cardiff, UK; 4 Neuroscience and Mental Health Research Institute, Cardiff, UK

**Keywords:** globus pallidus, hedonism, human values, medial forebrain bundle

## Abstract

Human values are abstract ideals that motivate behavior. The motivational nature of human values raises the possibility that they might be underpinned by brain structures that are particularly involved in motivated behavior and reward processing. We hypothesized that variation in subcortical hubs of the reward system and their main connecting pathway, the superolateral medial forebrain bundle (slMFB) is associated with individual value orientation. We conducted Pearson's correlation between the scores of 10 human values and the volumes of 14 subcortical structures and microstructural properties of the medial forebrain bundle in a sample of 87 participants, correcting for multiple comparisons (i.e.,190). We found a positive association between the value that people attach to hedonism and the volume of the left globus pallidus (GP).We then tested whether microstructural parameters (i.e., fractional anisotropy and myelin volume fraction) of the slMFB, which connects with the GP, are also associated to hedonism and found a significant, albeit in an uncorrected level, positive association between the myelin volume fraction within the left slMFB and hedonism scores. This is the first study to elucidate the relationship between the importance people attach to the human value of hedonism and structural variation in reward-related subcortical brain regions.

## Introduction

**Figure 1.**
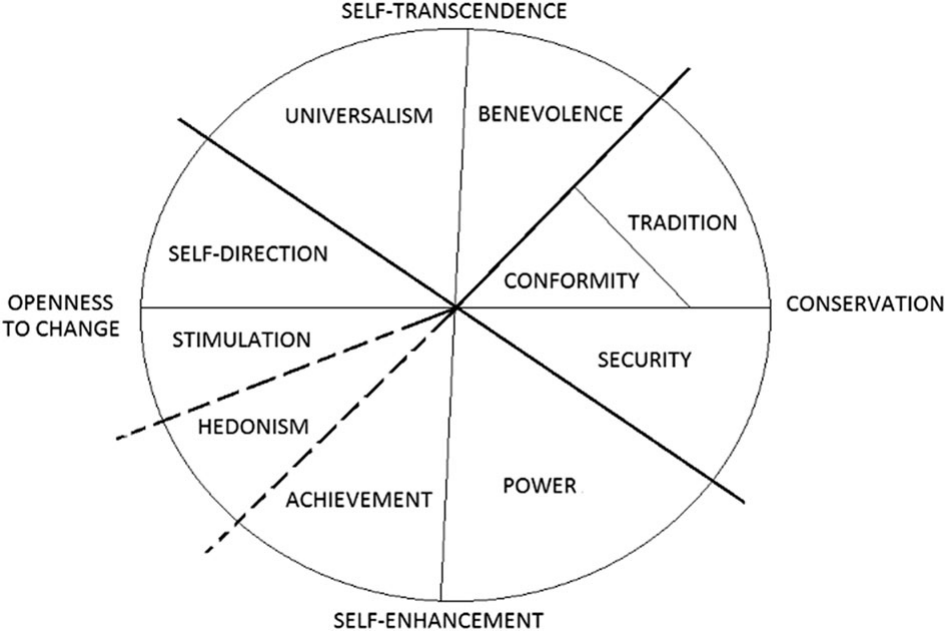
The circumplex structure of personal values

Human values motivate behavior and are a central element for the smooth functioning of societies. Psychologists initially conceptualized and assessed values as the kind of future activity that one wishes to perform (e.g., “social” values entail helping people and occupations such as social work, whereas “theoretical” values involve the search for truth and occupations such as scientific study). Subsequent work (Rokeach 1973) emphasize that values should be assessed as idealized standards that have an “ought” character, rather than a mere assessment of subtle likes and dislikes toward occupations, and emphasized the relative importance between different values. Schwartz's circular theory of values (Schwartz's et al. 1992, 2012) addresses 2 shortcomings of earlier value theories by identifying a culture-invariant set of values and by explaining how different human values relate to each. This model posits the existence of 10 values (Fig. 1), with each expressing certain motives. These motives are organized along 2 main dimensions. One dimension contrasts motives to promote the self (self-enhancement) against motives that transcend personal interests (self-transcendence), while the other dimension contrasts motives to follow the status quo (conservation) against motives to pursue personal intellectual and emotional interests in uncertain directions (openness).

(Modified from Schwartz 1992).

Knowledge of the neuroanatomical basis of human values is currently very limited. In a voxel-based morphometry (VBM) study using the Moral Foundation Questionnaire, Lewis et al. (2012) found a positive association between moral individualizing (conceptually similar to Schwartz's self-enhancement) and grey matter volume in the dorsomedial prefrontal cortex and a negative association with grey matter volume in the bilateral precuneus. Conversely, moral binding which is conceptually similar to Schwartz's conservation value dimensions, Boer and Fischer (2013), was positively associated with grey matter volume in the bilateral subcallosal gyrus of the frontal lobe. Apart from investigating human values, prior neuroanatomical studies also explored psychological variables related to human values, but distinct from them, including political attitudes, personality traits, and moral beliefs. Regarding the political attitudes, Kanai et al. (2011) found that greater liberalism was associated with increased grey matter volume in the anterior cingulate cortex and that greater conservatism was associated with increased volume of the right amygdala. However, the links between these political ideologies and values is unclear, because ideologies are related to multiple values (e.g., liberalism to the value dimensions of self-transcendence, self-enhancement) in ways that vary across nations and not to any particular values distinctly (e.g., Greenberg and Jonas 2003; Ashton et al. 2005). Gardini et al. (2009) investigated the link between grey matter volume and human value-related personality scores using the 3D Personality Questionnaire. First, reward dependence, similar to Schwartz's hedonism value, was negatively correlated with grey matter volume in the caudate nucleus and in the rectal gyrus, a part of the frontal lobe. Second, higher novelty seeking, an inclination similar to Schwartz's stimulation values, was associated with more grey matter volume in the right frontal and posterior cingulate regions. Third, there was a positive association between persistence, a tendency conceptually related to Schwartz's achievement value, and grey matter volume in the precuneus, paracentral lobule, and parahippocampal gyrus.

Overall, previous studies have found some structural brain correlates of values and value-related variables, but this work was focused exclusively on cortical regions. In addition, these dimensions are apparent in patterns of interrelations between the key values across samples from over 70 nations (Schwartz et al. 2012). This extensive cross-cultural support may imply that values express motives that have been evolutionarily conserved. If this is indeed the case, then these set of values is likely to be reflected in brain anatomy. In particular, such an evolutionarily conserved set of values is likely reflected in the brain structure. This can be assessed by investigating the association between the scores of these values and anatomical features of the brain (i.e., grey matter density and volume, white matter microstructure). In particular, the motivational nature of human values raises the possibility that they might be underpinned by certain subcortical brain regions that are particularly involved in motivated behavior and reward processing, a hypothesis that has never been directly tested. The putative reward system of the human brain is centered on hubs in the basal ganglia (striatum and globus pallidus [GP]) and includes the ventral tegmental area (VTA), prefrontal brain regions, and parts of the limbic system (Haber and Knutson 2010). These grey matter regions are structurally connected by white matter pathways. At the core of those connection pathways is the MFB that connects the VTA with the nucleus accumbens (NAcc), the medial and lateral orbitofrontal cortex and the dorsolateral prefrontal cortex (Bracht et al. 2014). There are 2 different branches of the MFB have been described previously: the inferomedial medial forebrain bundle (imMFB) and the superolateral medial forebrain bundle (slMFB) (Coenen et al. 2012). The latter may be of particular importance for reward processing. However, so far no study has investigated associations between the slMFB and values related to hedonism. It is the aim of this study to investigate if structural properties of basal ganglia and slMFB reflect human values. We examined correlations between human value scores, as assessed by the Schwartz value scale (Schwartz 1992), and the volume of 14 subcortical areas (left and right: GP, thalamus, caudate nucleus, putamen, hippocampus, amygdala, and NAcc) as well as the slMFB that connects many of these regions. We hypothesize a positive association between the hedonism score and the microstructural parameters within slMFB as well as the volume of the subcortical regions connected to slMFB.

## Materials and Methods

### Participants

Overall, 87 right-handed Caucasian university students between 19 and 42 (56 females; mean age = 23.97 ± 3.92 SD) participated in the study, all of whom were university students or graduates. Participants were informed that the study examined value-morality judgments with anatomical neuroimaging. Participants gave written informed consent, and the study was approved by the local ethics committee of Cardiff University. Human value scores beyond 3 standard deviations away from the mean were excluded from the analysis. We identified one such instance in 3 (hedonism, achievement, and conformity) out of 10 values.

### MRI Data Acquisition

All MRI data were acquired in the Cardiff University Brain Research Imaging Centre (CUBRIC) on a 3 T GE SignaHDx system (General Electric, Milwaukee, USA) equipped with an 8HR Brain parallel head coil for radio frequency transmission/reception.

#### Structural MRI

Anatomical high-resolution T1-weighted volume scans (1 mm^3^) were acquired using a fast spoiled gradient echo (FSPGR) 3-D sequence (TR = 7.849 ms; TE = 2.984 ms; field of view = 256 × 256 mm^2^; voxel size = 1 × 1 × 1 mm^2^).

#### Multicomponent Relaxometry

Myelin measures were derived using Multi-Component Driven Equilibrium Single Pulse Observation of T1 and T2 (mcDESPOT) (Deoni et al. 2008). The acquisition consists of Spoiled Gradient Recall (SPGR) images across 8 flip angles, one inversion recovery SPGR (IR-SPGR) and steady-state free precession (SSFP) images across 8 flip angles and 2 phase-cycling angles. A total of 25 images were acquired for each subject. All images were acquired in sagittal orientation with a slice matrix of 128 × 128 (1.72 × 1.72 mm^2^ resolution) with a minimum of 88 slices (slice thickness = 1.7 mm). Sequence-specific parameters were: SPGR: TE = 2.112 ms, TR = 4.7 ms, flip angles = 3°, 4°, 5°, 6°, 7°, 9°, 13° and 18°. IR-SPGR: TE = 2.112 ms, TR = 4.7 ms, IR = 450 ms, flipangle = 5°. SSFP: TE = 1.6 ms, TR = 3.2 ms, flip angles of 10.59°, 14.12°, 18.53°, 23.82°, 29.12°, 35.29°, 45°, and 60°; and phase-cycling angles of 0° and 180°.

#### Diffusion-Weighted Imaging

Diffusion MRI comprising a cardiac-gated diffusion-weighted spin-echo echo-planar imaging sequence was used to acquire high angular resolution diffusion-weighted images (HARDI). A total of 30 gradient orientations (*b* = 1200 s/mm^2^) and 3 unweighted (*b* = 0 s/mm^2^) images were acquired with the following parameters: TE = 87 ms, 60 slices, slice thickness = 2.4 mm, FoV = 230 × 230 mm^2^, acquisition matrix = 96 × 96, resulting in data acquired with a 2.4 × 2.4 × 2.4 mm^3^ isotropic resolution following zero-filling to a 128 × 128 in-plane matrix for the fast Fourier transform. The final image resolution was therefore 1.8 × 1.8 × 2.4 mm^3^.

### Preprocessing

#### Structural MRI

Cortical reconstruction and volumetric segmentation of 14 subcortical areas (Supplementary Material 1, left and right: GP, thalamus, caudate nucleus, putamen, hippocampus, amygdala, and NAcc) was performed with FreeSurfer image analysis software v4.4.0, which is documented and freely available for download on-line (surfer.nmr.mgh.harvard.edu). All correlation analyses were performed on the Software Package for Statistical Analysis (SPSS for Windows version 19.0). As subcortical volume may be confounded by confounded by age, gender and overall intracranial volume (ICV) we controlled for these effects in the regression analysis ICV derived from VBM8 (Gaser 2009), SPM8, (http://www.fil.ion.ucl.ac.uk/spm/software/spm8).

#### Multicomponent Relaxometry

All images were linearly coregistered to the 13° SPGR image to correct for subject motion. Non-brain tissue was removed using a mask computed with the BET algorithm (Smith, 2002). Registration and brain masking were performed with FSL (http://www.fmrib.ox-.ac.uk/fsl/). The images were then corrected for B1 inhomogeneities and off-resonance artefacts, using maps generated from the IR-SPGR and 2 phase-cycling SSFP acquisitions, respectively. The 3-pool mcDESPOT algorithm was then used to identify a fast (water constrained by myelin) and slow (free-moving water in intra- and extra-cellular space) components of the T1 and T2 times, and a non-exchanging free-water component (Deoni et al. 2013). The fast water fractionwas taken as a map of the myelin-water fraction.

#### Diffusion MRI

Data were analyzed using *ExploreDTI* 4.8.3 (Leemans et al. 2009). Eddy-current induced distortion and motion correction was performed using an affine registration to the non-diffusion-weighted B_0_-images, with appropriate re-orienting of the encoding vectors (Leemans and Jones 2009). Field inhomogeneities were corrected for using the approach of (Wu et al. 2008). The diffusion-weighted images (DWIs) were non-linearly warped to the T_1_-weighted image using the fractional anisotropy (FA) map, calculated from the DWIs, as a reference. Warps were computed using Elastix (Klein et al. 2010) using normalized mutual information as the cost function and constraining deformations to the phase-encoding direction. The corrected DWIs were therefore transformed to the same (undistorted) space as the T_1_-weighted structural images. A single diffusion tensor model was fitted to the diffusion data in order to compute quantitative parameters such as FA (Basser et al. 1994). A correction for free water contamination of the diffusion tensor based estimates was applied, before sampling diffusion properties (e.g., FA) along the fornix and the PHC (Pasternak et al. 2009; Metzler-Baddeley et al. 2012). The FA, radial, axial, and mean diffusivities (RD, AD, MD) was then computed from the DT.

#### Tractography of the Superolateral Medial Forebrain Bundle

Whole brain tractography was performed using the damped Richardson-Lucy algorithm (Dell'acqua et al. 2010), and an algorithm similar to that described by (Basser et al. 1994). Termination criteria were an angle threshold > 45° and FA < 0.2. The slMFB was reconstructed as described in (Bracht et al. 2015). One horizontal ROI was placed surrounding the VTA. Anatomical borders were laterally the substantia nigra, anteriorly the mammillary bodies and posteriorly the red nucleus (Nieuwenhuys et al. 2008). A second ROI was drawn surrounding caudate and putamen on a coronal section at the height of the NAcc. Due to the particular interest in the role of the MFB in reward processing, the focus was placed on segments of the slMFB dorsal to the VTA including projections from the VTA to NAcc, GP, hypothalamus and the OFC, core regions of reward processing.

## Human Values

Participants completed the Schwartz value survey (SVS; Schwartz 1992), which was administered in the laboratory before the scanning session. Participants were asked to rate how important each of 56 values is as a guiding principle in their lives, using a quasi-bipolar 9-point scale ranging from −1 (opposed to my values), 0 (not important), 4 (important), to 7 (of supreme importance). Examples of SVS items are as follows: “Equality: Equal opportunity for all” (Universalism); “Pleasure: Gratification of desires” (Hedonism); “Obedient: Dutiful meeting obligations” (Conformity). The average score across the 56 items was then calculated and subtracted from each of the 56 initial raw scores. Schwartz recommends this procedure to help control for superfluous individual variations in rating styles (e.g., Schwartz 1992). The raw value distribution of the Schwartz Value Survey can be seen in Supplementary Material 3.1. The internal consistency, as measured by the Cronbach's alpha, of these indices was moderate to good (Supplementary Material 3.2). Moreover, to validate Schwartz's hypothesized circular structure in our sample, we conducted 2 Multidimensional Scaling (MDS) analyses (Bilsky et al. 2011). The first analysis plotted the 56 value items, and the second analysis plotted the 10 higher-order values; both analyses used the respective correlation matrix to plot the values in a 2D space. The first analysis yielded S-stress  = 0.167 and Stress I = 0.274, while the second one yielded S-Stress = 0.032 and a Stress-I = 0.115. The stress value is an index of how well the data fit the hypothesized configuration; higher stress values signify a poorer configuration. The stress values and the patterns in the MDS (Supplementary Material 3.3) supported to a large extent to the structure hypothesized by Schwartz (1992).

We conducted correlations between the human value scores (i.e., 10 values) and the residual scores of all 14 subcortical measures as well as 5 medial forebrain bundle microstructural measures of (after regressing out the age, gender and intracranial volume or overall microstructural properties). The Bonferroni correction which was calculated using the R^TM^ 3.0.2. software package using the code, p.adjust (p, method = “bonferroni”). Bootstrapped confidence intervals (95%) were computed using AMOS software. We conducted an internal replication analysis of our primary finding by calculating correlations in 2 randomly selected subsets of our sample. The volume of left GP significantly predicted hedonism scores in both sub-samples, *r*(42) = 425, *P* = 0.005; *r*(43) = 0.353, *P* = 0.02.

## Results

First, we compared the volume of the left to the right GP. Left GP (*M* = 1667 mm^3^) was significantly larger (*t*(86) = *P* < 0.00001) than the right (*M* = 1490 mm^3^), as reported previously (Kooistra and Heilman 1988).

**Figure 2.**
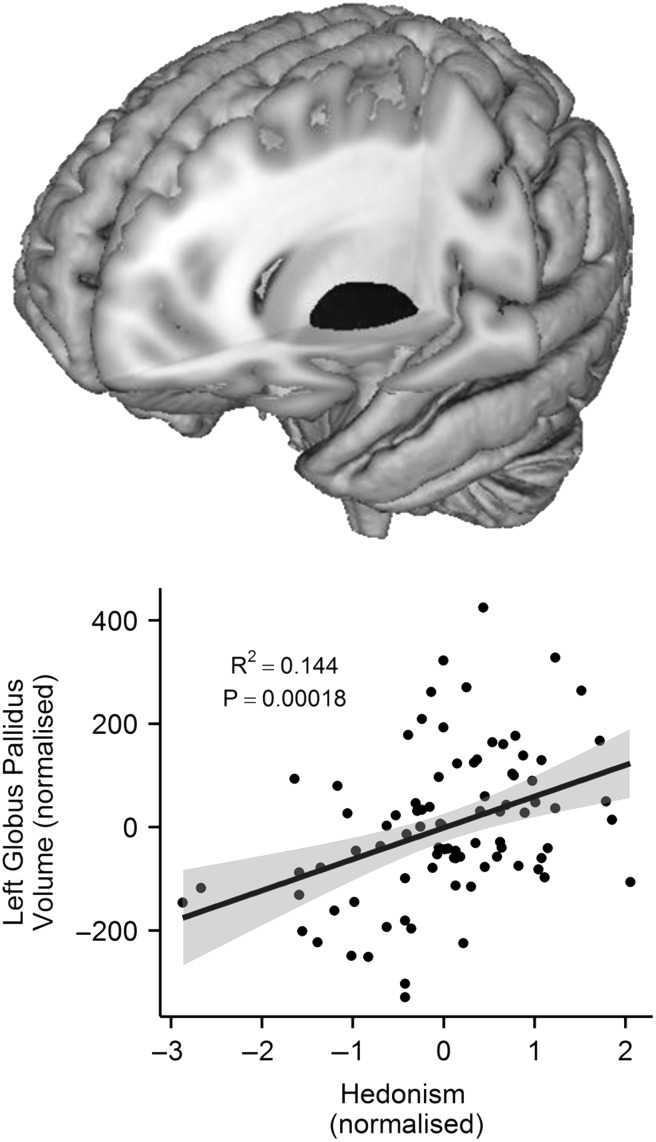
A 3D-mesh of the left GP (above) and a scatter-plot of the correlation (*r*(84) = 0.393, *P*_*(BONF)*_ = 0.035) between the volume of the left GP (i.e., the residual scores with age, gender and intracranial volume regressed out) (*x*-axis) and the standardized hedonism scores (*y*-axis). Each dot represents one participant. The volume of the left GP significantly predicts hedonism scores. Grey shading around the regression line represents 95% confidence interval.

We then investigated the association between the subcortical volume of 14 structures, as well as 5 microstructual properties of the slMFB, and the 10 human values. After a robust (Bonferroni) multiple comparison correction (i.e., 10 values x [14 subcortical structures + 5 slMFB measures]), we found that people who rated hedonism as important in their life had a larger GP in the left hemisphere, *r*(84) = 0.393, *P*_(BONF)_ = 0.035 (Fig. 2) (*P*_(UNCORR)_ = 0.000182). The robustness of the association between left GP volume and hedonism score was confirmed by internal replication (Material, Human Values section) and bootstrapping (95% confidence intervals: *r* = 0.393, lower = 0.232, upper = 0.537, *P* = 0.002 [*N* = 1000]; *r* = 0.398, lower = 0.241, upper = 0.540 , *P* < 0.0005 ([*N* = 5000]). The next highest correlation between any value and any of the examined brain areas was observed for stimulation and the volume of the left GP, *r*(85) = 0.231, *P*_(UNCORR)_ = 0.032, although this correlation did not reach the corrected significance level. The relationship between hedonism score and left GP when including the outlier scores was *r*_s_(85) = 0.398 *P*_(UNCORR)_ = 0.000134; *P*_(BONF)_ = 0.025. In addition, similar to left GP, we found that the right GP is also positively related to hedonism (*r*(84) = 0.218, *P*_(UNCORR)_ = 0.44) but this association did not survive the Bonferroni correction. Because of this relative difference between left and right, we also examined whether the left minus right GP volume may be associated to hedonism. Indeed, there is a positive association between the left minus right GP volume and hedonism *r*(84) = 0.266, *P*_(UNCORR)_ = 0.013 (the correlation coefficient can be seen in Supplementary Material 4. For completeness, the association between the rest of subcortical measures and hedonism can be seen in Supplementary Material 2. This pattern of associations suggests that hedonism is specifically related to GP and not to any other subcortical structure.

Although none of the slMFB and hedonism survived the aforementioned Bonferroni correction, we present here the strongest slMFB associations to hedonism. There was a positive association between the myelin volume fraction of the left slMFB, *r*(79) = 0.312, *P*_(UNCORR)_ = 0.005) (Supplementary Material 2) and right slMFB, *r*(79) = 0.266, *P*_(UNCORR)_ = 0.017, (corrected for overall whole brain myelin volume fraction, age, and gender) and hedonism. These associations still hold, at an uncorrected level, when correcting for overall white matter restricted myelin volume fraction, age and gender (left, r(79) = 0.245, *P*_(UNCORR)_ = 0.028; right, *r*(79) = 0.220, *P*_(UNCORR)_  = 0.049). For completeness, we present the association between hedonism and FA, MD, RD, and L1 of left and right MFB (Supplementary Material 2).

## Discussion

The present study investigated the neural representation of the motivational nature of human values by testing for associations between the scores of 10 values and the volume of 14 subcortical regions as well as the myelin volume fraction of slMFB. Our results demonstrate, for the first time, a direct association between the value that people attach to hedonism and the volume of a specific brain structure, the left GP as well as the myelin volume fraction of slMFB.

The slMFB has been previously associated to hedonic-related mechanisms (Bracht et al. 2016). Specifically Bracht et al. (2014) found that mean-FA within the MFB was correlated negatively with depression scale rating scores. In contrast to the direction of this finding, Bracht et al. (2015) found that hedonic capacity was correlated negatively with mean FA of the left slMFB. Here, we calculated both FA and MWF of the slMFB but only the MWF showed a clear trend to hedonism. Compared with FA, MWF is considered a more biologically interpretable proxy of white matter myelination. Myelin enables faster and more efficient propagation of action potentials along axonal pathways, which in turn can contribute to faster information processing capabilities (Turken et al. 2008).Specifically, it provides more information about the tissue composition of white matter connections, independent of volume (to which other parameters such as axon diameter and inter-axonal space can contribute).

The observed association between structural variability in the slMFB and hedonism complements previously established links between fronto-striatal and limbic-striatal microstructural connectivity, striatal reward-related processing and personality traits (Cohen et al. 2009). For example, individual differences in novelty seeking were associated with the microstructural strength of connections between hippocampus, ventral striatum and midbrain while the microstructural strength of the tracts between prefrontal cortex and striatum explained individual differences in reward dependence (Cohen et al. 2009). Furthermore, novelty seeking and the reward dependence temperaments were associated with fronto-striatal fiber connectivity (Lei et al. 2014). Moreover, in a DTI-fMRI study, FA of cortico-striatal fiber tracts was related to NAcc reward-related activation (Koch et al. 2013).

GP is a relatively large subcortical structure which is in a dorsal and a ventral segment. The dorsal segment is particularly implicated in motor control, while the ventral segment, which receives input from the NAcc, has been involved in hedonic-related processing (see below). The GP region in the present study includes both dorsal and ventral GP segments (Supplementary Material 1). The GP is a central node not only in the direct and indirect pathways that govern motor control (underpinned mainly by the dorsal segment), but also in the “executive” and “limbic” circuits of the basal ganglia (Rodriguez-Oroz et al. 2009). The limbic circuit, in particular, has been implicated in motivated behavior. This circuit originates from the projections of the ventral striatum to the GP and continues to the thalamus. Inferences from the size of a region onto its function are limited but one might speculate that a larger volume of the GP, presumably reflecting a higher number of neurons and/or more neuropil, would result in hypermotivated states, such as those associated with hedonism. Previous lesion studies have indeed implicated the GP in reward activation. For example, Rochat et al. (2013) conducted a voxel-based lesion-symptom mapping, which showed that damage to the GP was associated with poorer reward sensitivity. In addition, a case study described a patient who developed severe anhedonia after he sustained bilateral GP lesions (Miller et al. 2006).

With respect to laterality, it has been previously demonstrated (Kooistra and Heilman 1988) that the left GP is often larger than the right GP and this is what we found in our study. A previous study (Glick, Ross & Hough, 1982) on neurochemical asymmetries showed a leftward asymmetry in dopamine levels in GP. A later study (Lauterbach et al. 1997) on focal subcortical lesion patients, suggested that depression onset may be caused specifically after left posterior GP lesions potentially by disturbing basal ganglia thalamocortical mood circuits. Taken together, these structural, neurochemical and lesion laterality studies suggest that the left GP is a subcortical structure particularly associated with the maintenance of healthy reward-related mechanisms.

Functional imaging studies have also supported the role of GP in reward sensitivity. A recent meta-analysis (Arsalidou et al. 2013) revealed the involvement of the left lateral GP in reward processing and of the left medial GP in tasks that required eliciting or judging emotions. Lastly, prior animal work supports a hedonic function for networks involving the GP. For example, Ho and Berridge (2013) investigated the neuronal connections of the orexin terminals in the posterior half of ventral pallidum, a region in close proximity to opioidergic hedonism “hotspots”. By injecting orexin-A into this region, they enhanced the hedonic impact of sucrose, as assessed via affective taste reactivity (Ho and Berridge 2013).

Our study, which is the first to demonstrate a robust role for the GP in hedonism in healthy humans, thus fits with neuropsychological and animal models of “wanting” and “liking” (Smith et al. 2011). Our finding has potential implications for both clinical and social neuroscience. Individual variation in GP volume might partly determine susceptibility to hedonic deficits associated with addiction or mood disorders. More broadly, it is also possible that this variation might contribute to our understanding of the role of impulsive, hedonistic inclinations in a number of difficult societal behavior change issues (see Maio et al. (2008) for a review), such as attempts to attenuate increasing levels of obesity, damage to the environment, and antisocial behavior.

Despite the fact that our volumetric findings are merely correlational and cannot demonstrate that larger GP increases the subjective importance of hedonism as a human value, this novel hypothesis can be addressed in developmental studies testing whether inter-individual variation in left GP volume across the lifespan is associated with the subjective importance of the value of hedonism. From the functional neuroanatomical perspective, it has been previously suggested that larger grey matter volume results in better computational efficiency in that region (Kanai et al. 2011).

Another important question is whether larger GP volume leads to high importance of hedonism or vice-versa. Previous behavioral genetics studies (Shermer et al. 2008, 2011; Zacharopoulos et al. 2016) demonstrated that human values may have genetic aetiology. However, the behavioural genetics of human values is a relatively new field and thus knowledge on the specific genetic markers for particular human values, such as hedonism, are still poorly understood. The findings of the present study provide the first endophenotype (i.e., volumetric variation in the GP) that may mediate the association between specific genetic markers and hedonism. The direction of the GP-hedonism link can be determined in future mediational studies. In particular, if the relationship between specific genetic components and the relative importance of hedonism is mediated by the volumetric variation of left GP, while the relationship between the same genetic components and volumetric variation of left GP is not mediated by hedonism, then one could make the case for left GP volume causes changes in the subjective value of hedonism.

In sum, the present research (a) demonstrated a strong positive association between the volume of left GP and the human value of hedonism, and (b) extended prior findings on the association between hedonic processes and the microstructural properties of slMFB. Together, these results provided the first direct association between the importance people attach to the human value of hedonism and structural variation in reward-related subcortical brain regions. This provides a novel source of evidence pertinent to affective neuroscience research on reward-related deficits, such as the anhedonia in major depression.

## Supplementary Material

Supplementary material can be found at: http://www.cercor.oxfordjournals.org/

## Funding

This study was supported by the National Centre for Mental Health (NCMH) at Cardiff University, with funds from the National Institute for Social Care and Health Research (NISCHR), Welsh Government, Wales (Grant no. BR09), and by Grant MR/K004360/1 from the Medical Research Council (MRC) and by the MRC Centre for Neuropsychiatric Genetics and Genomics (G0800509).

## Supplementary Material

Supplementary Data
