# Supplementary material for: A Hedonism Hub in the Human Brain
Source: Cereb Cortex. 2016 Sep 19;26(10):3921–7. doi: 10.1093/cercor/bhw197 (PMC5028005; doi:10.1093/cercor/bhw197)
Supplement: Supplementary Data [file supp_26_10_3921__index.html]

A Hedonism Hub in the Human Brain — Supplementary Data 

# A Hedonism Hub in the Human Brain

## Supplementary Data

Supplementary Data

- Supplementary Data - tif file
- Supplementary Data - docx file
- Supplementary Data - docx file
- Supplementary Data - tif file
