# Supplementary figures and images for: A Hedonism Hub in the Human Brain
Source: Cereb Cortex. 2016 Sep 19;26(10):3921–7. doi: 10.1093/cercor/bhw197 (PMC5028005; doi:10.1093/cercor/bhw197)

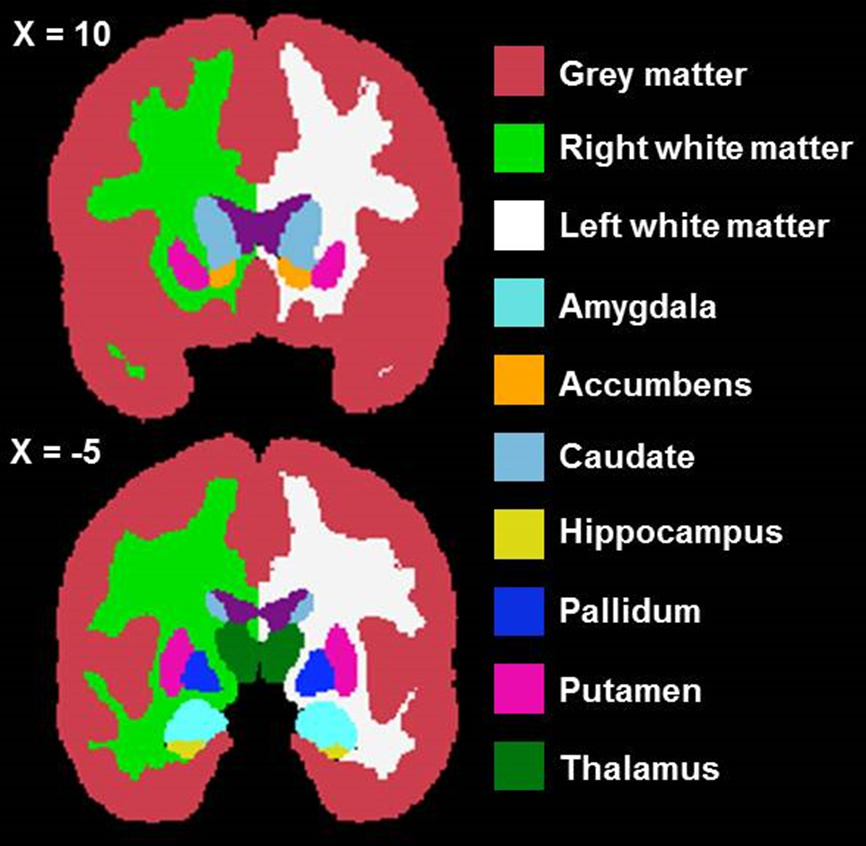

Supplement: Supplementary Data [file supp_bhw197_Supplementary_Material1.tif]

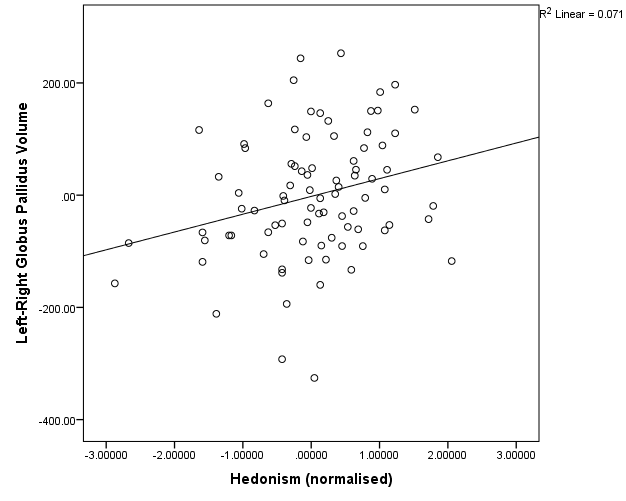

Supplement: Supplementary Data [file supp_bhw197_Supplementary_Material4.tif]
