# Supplementary material for: A Hedonism Hub in the Human Brain
Source: Cereb Cortex. 2016 Sep 19;26(10):3921–7. doi: 10.1093/cercor/bhw197 (PMC5028005; doi:10.1093/cercor/bhw197)
Supplement: Supplementary Data [file supp_bhw197_Supplementary_Material2.docx]

**Supplementary Material 2**. Correlation coefficients between the ten human values (CO=Conformity, TR=Tradition, BE=Benevolence, UN=Universalism, SD= Self-direction, ST=Stimulation, HE=Hedonism, AC=Achievement, PO=Power, SE=Security) and the 14 subcortical structures (left and right: globus pallidus, thalamus, caudate nucleus, putamen, hippocampus, amygdala, nucleus accumbens), as well as and several fractional anisotropy and myelin volume fraction indices *(*wm: restricted to white matter mask, wb=whole brain, #: controlling for age, gender and overall fractional anisotropy within the white matter mask, +: controlling for age, gender, and overall fractional anisotropy).

|  | CO | TR | BE | UN | HE | SD | ST | AC | | PO | SE |
| --- | --- | --- | --- | --- | --- | --- | --- | --- | --- | --- | --- |
|  |  |  |  |  |  |  |  |  | |  |  |
| L_Thalamus | 0.033 | -0.011 | 0.064 | -0.127 | 0.105 | -0.054 | 0.021 | -0.026 | | -0.091 | 0.15 |
| R_Thalamus | 0.032 | -0.055 | -0.084 | -0.119 | 0.192 | -0.088 | 0.114 | 0.027 | | 0.041 | 0.109 |
| L_Caudate | -0.088 | -0.08 | -0.052 | 0.078 | 0.149 | 0.055 | 0.041 | | 0.145 | -0.113 | -0.036 |
| R_Caudate | -0.035 | -0.038 | 0.002 | 0.129 | 0.144 | 0.021 | 0.03 | | 0.018 | -0.099 | -0.08 |
| L_Putamen | -0.076 | -0.002 | -0.106 | -0.059 | 0.053 | -0.101 | 0.142 | | -0.005 | 0.188 | -0.02 |
| R_Putamen | -0.037 | 0.033 | -0.129 | -0.025 | -0.005 | -0.144 | 0.13 | | -0.079 | .217* | 0.044 |
| L_Pallidum | 0 | -0.182 | -0.021 | -0.152 | .393** | 0.036 | .231* | | 0.083 | -0.058 | 0.002 |
| R_Pallidum | -0.1 | -0.152 | 0.085 | -0.125 | .218* | 0.001 | 0.052 | | 0.014 | 0.039 | -0.035 |
| L_Hippocampus | 0.042 | 0.042 | -0.062 | -0.003 | -0.021 | -0.105 | 0.096 | | -0.007 | -0.004 | 0.014 |
| R_Hippocampus | -0.035 | 0.073 | -0.023 | 0.024 | 0.072 | -0.134 | 0.183 | | -0.048 | -0.004 | 0.082 |
| L_Amygdala | -0.142 | -0.029 | -0.068 | 0.073 | -0.124 | -0.069 | 0.033 | | 0.036 | 0.205 | -0.115 |
| R_Amygdala | 0.084 | -0.002 | -0.081 | -0.013 | -0.15 | 0.014 | -0.038 | | 0.077 | 0.045 | -0.046 |
| L_Accumbens | 0.075 | 0.166 | 0.061 | -0.13 | -0.09 | -0.038 | -0.056 | | -0.053 | 0.012 | -0.016 |
| R_Accumbens | -0.092 | -0.079 | -0.115 | 0.018 | 0.114 | 0.072 | 0.055 | | 0.179 | 0.091 | -0.182 |
| L_MVF_wm | 0.061 | 0.09 | 0.035 | -0.198 | .245* | -0.163 | 0.06 | | 0.134 | 0.038 | 0.032 |
| R_MVF_wm | -0.058 | 0.012 | 0.153 | -0.027 | .220* | -0.089 | 0.041 | | 0.011 | -0.004 | 0.049 |
| L_MVF_wb | 0.013 | 0.021 | 0.066 | -0.2 | .312** | -0.142 | 0.051 | | 0.108 | 0.03 | 0.145 |
| R_MVF_wb | -0.066 | -0.049 | 0.122 | -0.064 | .266* | -0.067 | 0.041 | | 0.023 | -0.013 | 0.155 |
| L_FA_wm# | 0.007 | 0.106 | -0.001 | -0.176 | 0.107 | -0.042 | 0.144 | | 0.145 | 0.001 | -0.062 |
| R_FA_wm# | -0.042 | 0.091 | 0.105 | -0.027 | 0.056 | -0.031 | 0.112 | | -0.014 | -0.096 | 0.006 |
| L_FA_wb+ | -0.037 | 0.087 | 0.007 | -0.211 | 0.136 | -0.051 | 0.091 | | 0.157 | 0.034 | 0.003 |
| R_FA_wb+ | -0.062 | 0.072 | 0.098 | -0.058 | 0.071 | -0.032 | 0.068 | | 0 | -0.075 | 0.058 |
| * p< 0.05, ** p< 0.01 (2-tailed). | | |  |  |  |  |  | |  |  |  |
