# Supplementary material for: A Hedonism Hub in the Human Brain
Source: Cereb Cortex. 2016 Sep 19;26(10):3921–7. doi: 10.1093/cercor/bhw197 (PMC5028005; doi:10.1093/cercor/bhw197)

**Supplementary Material 3**.

3.1. Raw value distribution of all 10 human values

|  | | | | | |
| --- | --- | --- | --- | --- | --- |
|  | N | Minimum | Maximum | Mean | Std. Deviation |
| Conformity | 86 | .75 | 7.00 | 4.4913 | 1.20321 |
| Tradition | 86 | .17 | 5.67 | 2.5891 | 1.26664 |
| Benevolence | 87 | 2.50 | 6.63 | 5.0848 | .85643 |
| Universalism | 87 | 2.44 | 6.89 | 4.9757 | 1.01120 |
| Self- Direction | 87 | 3.17 | 7.00 | 5.3257 | .86150 |
| Stimulation | 87 | 1.33 | 7.00 | 4.8199 | 1.36948 |
| Hedonism | 87 | 2.00 | 7.00 | 5.5517 | 1.08648 |
| Achievement | 86 | 2.40 | 7.00 | 5.1186 | 1.02228 |
| Power | 87 | -.80 | 6.60 | 2.5011 | 1.41318 |
| Security | 87 | 1.29 | 6.71 | 4.7274 | 1.03237 |
| Valid N (listwise) | 85 |  |  |  |  |

3.2. Cronbach’s alpha for each of the 10 values

| Value | Number of items | Cronbach’s α |
| --- | --- | --- |
| Universalism | 7 | .76 |
| Benevolence | 9 | .76 |
| Tradition | 6 | .63 |
| Conformity | 4 | .63 |
| Security | 6 | .68 |
| Power | 5 | .79 |
| Achievement | 6 | .67 |
| Hedonism | 2 | .74 |
| Stimulation | 3 | .79 |
| Self-direction | 6 | .65 |

3.3. Multidimensional Scaling analyses

3.3.1. Multi-Dimensional Scaling: Multidimensional scaling analysis of 56 items.


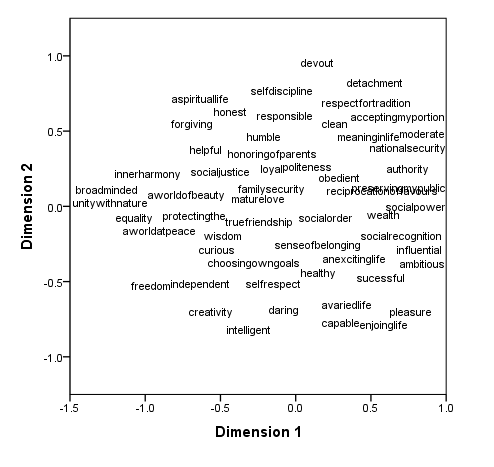


3.3.2. Multi-Dimensional Scaling: Multidimensional scaling analysis of 10 values.


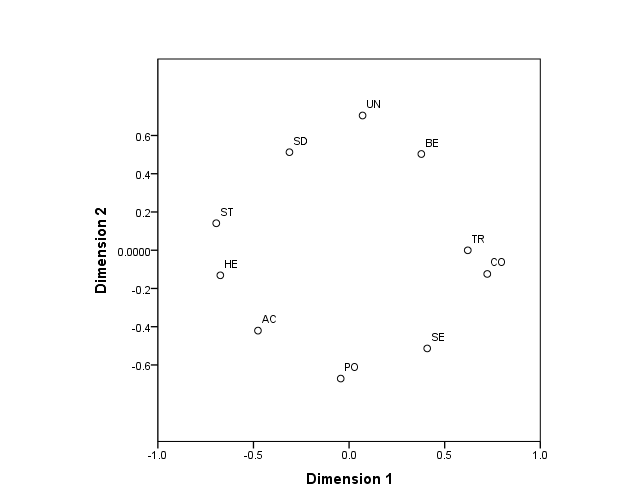

Supplement: Supplementary Data [file supp_bhw197_Supplementary_Material3.docx]
